# Hesperidin enhances intestinal barrier function in Caco‐2 cell monolayers via AMPK‐mediated tight junction‐related proteins

**DOI:** 10.1002/2211-5463.13564

**Published:** 2023-02-07

**Authors:** Ha‐Young Park, Jin‐Hee Yu

**Affiliations:** ^1^ Advanced Radiation Technology Institute Korea Atomic Energy Research Institute Jeongeup Korea

**Keywords:** AMP‐activated protein kinase, Caco‐2 cells, hesperidin, paracellular transport, tight junction

## Abstract

The intestinal epithelium is a single‐cell layer on the mucosal surface that absorbs food‐derived nutrients and functions as a barrier that protects mucosal integrity. Hesperidin (hesperetin‐7‐rhamnoglucoside) is a flavanone glycoside composed of the flavanone hesperetin and the disaccharide rutinose, which has various physiological benefits, including antioxidative, anti‐inflammatory, and antiallergic effects. Here, we used human intestinal Caco‐2 cell monolayers to examine the effect of hesperidin on intestinal barrier function. Hesperidin‐treated Caco‐2 cell monolayers displayed enhanced intestinal barrier integrity, as indicated by an increase in transepithelial electrical resistance (TEER) and a decreased apparent permeability (P_app_) for fluorescein. Hesperidin elevated the mRNA and protein levels of occludin, MarvelD3, JAM‐1, claudin‐1, and claudin‐4, which are encoded by tight junction (TJ)‐related genes. Moreover, hesperidin significantly increased the phosphorylation of AMP‐activated protein kinase (AMPK), indicating improved intestinal barrier function. Thus, our results suggest that hesperidin enhances intestinal barrier function by increasing the expression of TJ‐related occludin, MarvelD3, JAM‐1, and claudin‐1 via AMPK activation in human intestinal Caco‐2 cells.

## Graphical abstract

Hesperidin is potentially effective in improving intestinal barrier function. Hesperidin enhances the intestinal barrier integrity, as indicated by an increase in transepithelial electrical resistance (TEER) and a decreased apparent permeability (P_app_) for fluorescein. In addition, hesperidin increases the expression of tight junction‐related occludin, MarvelD3, JAM‐1, and claudin‐1 via AMPK activation in human intestinal Caco‐2 cells.

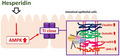

**Abbreviations**
AMP: adenosine monophosphate
AMPK: AMP‐activated protein kinase
CaMKK: Ca^2+^/calmodulin‐dependent protein kinase kinase
CCK‐8: Cell Counting Kit‐8
DMEM: Dulbecco's modified Eagle's medium
DMSO: dimethylsulfoxide
FBS: fetal bovine serum
HBSS: Hanks's balanced salt solution
HEPES: 4‐(2‐hydroxyethyl)‐1‐piperazine ethanesulfonic acid
JAM‐1: junctional adhesion molecule‐1
LKB1: liver kinase B1
Marvel: MAL and related proteins for vesicle trafficking and membrane link
P_app_
: apparent permeability
RT‐PCR: reverse transcription PCR
TAK‐1: transforming growth factor‐β‐activated kinase
TBS‐T: Tris‐buffered saline; 0.05% Tween 20
TEER: transepithelial electrical resistance
TJ: tight junction
ZO: zonula occludens

## Introduction

The intestinal epithelium is a single‐cell layer on the mucosal surface that absorbs food‐derived nutrients and functions as a barrier that protects the mucosal integrity, which physically prevents the permeation of harmful substances, such as bacteria‐derived components and antigenic agents from the external environment [1, 2]. Impairment of the intestinal barrier integrity allows the passage of excluded substances, which induce inflammatory immune responses. Therefore, intestinal health and diseases are closely linked to the intestinal barrier function and integrity, which are regulated by the mucous layer and tight junction (TJ) structures of epithelial cells [2]. Intestinal TJ structures are composed of multiple essential proteins, including transmembrane proteins, such as occludin, junctional adhesion molecule‐1 (JAM‐1), and claudins, along with intracellular proteins, such as zonula occludens (ZO) and cingulin [3, 4]. The intestinal barrier integrity and function are dynamically regulated by multiple factors, including pathogens and dietary nutrients [2]. Impairment of the epithelial barrier function is associated with various gastrointestinal disorders. However, nutraceutical factors that enhance intestinal epithelial differentiation can reinforce the epithelial barrier function and gut health, indicating that intestinal barrier integrity and epithelial permeability are essential for a healthy gut. The transepithelial electrical resistance (TEER) is predictive of epithelial barrier integrity and related to the expression of TJ proteins and their arrangement in epithelial cells [5]. Numerous studies support the beneficial effects of polyphenols on intestinal barrier enhancement, including intestinal barrier integrity and TJ formation. Recent studies have revealed that the activities of various intracellular signaling molecules modulate the integrity of TJ [6, 7]. TJ protein expression and connection with the actin cytoskeleton, which affects TJ permeability, are dynamically regulated by numerous intracellular signaling molecules, such as mitogen‐activated protein kinases, protein kinase C, and phosphatidylinositol 3‐kinase [8]. Furthermore, polyphenols can activate adenosine monophosphate (AMP)‐activated protein kinase (AMPK), an energy sensor that adjusts TJ and epithelial permeability [1]. Hesperidin (hesperetin‐7‐rhamnoglucoside) is a flavanone glycoside comprised of the flavanone hesperetin and the disaccharide rutinose. It is a common constituent of citrus fruits and is often referred to as vitamin P. There is a great interest in hesperidin because of its various physiological benefits, including its antioxidative, anti‐inflammatory, and antiallergic effects [9, 10, 11, 12]. However, the beneficial effects of hesperidin on intestinal epithelial differentiation and barrier function are poorly understood, and the mediation of hesperidin‐associated effects by AMPK has been rarely explored. The objective of this study was to assess whether hesperidin has a beneficial effect on the expression of intestinal barrier‐related TJ genes and to further clarify the role of AMPK in linking hesperidin to the intestinal barrier function.

## Materials and methods

### Materials

Hesperidin, fluorescein sodium salt, and dimethylsulfoxide (DMSO) were purchased from Sigma‐Aldrich (St. Louis, MO, USA). Dulbecco's modified Eagle's medium (DMEM) and fetal bovine serum (FBS) were obtained from Hyclone Laboratories Inc (Logan, UT, USA). Penicillin–Streptomycin, MEM Non‐Essential Amino Acids (NEAA), and 0.25% Trypsin/EDTA were purchased from GIBCO (Gaithersburg, MD, USA). MITO+™ serum extender, basal medium for seeding, and enterocyte differentiation medium for Caco‐2 cell monolayer cultures were purchased from BD Biosciences (San Jose, CA, USA). Other reagents were analytical grade and used without further purification.

### Cell culture

Caco‐2 cells (Korean Cell Line Bank, Seoul, Korea) were cultivated and maintained under standard cell culture conditions as described previously [13]. Caco‐2 cells were cultured at 37 °C under a 5% CO_2_ atmosphere in DMEM supplemented with 10% FBS, 1% NEAA, 100 U·mL^−1^ penicillin, and 100 μg·mL^−1^ streptomycin. The medium was replaced every other day, and cells were detached with 0.25% Trypsin/EDTA when they reached 70–80% confluence. Caco‐2 cells used in this study were between passages 40 and 60. For the transport study, cells seeded at a density of 4 × 10^5^ cells·mL^−1^ were grown in a cell culture insert (PET membrane, membrane 0.9 cm^2^, 1.0 μm pore size; Corning, Glendale, AZ, USA) coated with type I collagen (collagen gel culturing kit, Cellmatrix type I‐A, Wako, Osaka, Japan). For 48 h, the cells were cultured in a basal medium for seeding with MITO+™ serum extender as a supplement. Then, the medium was changed to enterocyte differentiation medium containing MITO+™ serum extender, and incubation was continued for 72 h to form monolayers. The medium was refreshed every day. TEER values were measured with the Multi‐Channel Voltage/Current EVE 4000 system (World Precision Instruments, Sarasota, FL, USA) and used to confirm the integrity of the monolayers (Fig. S2).

### Cell treatment

Caco‐2 cell monolayers were pretreated in DMEM supplemented with 10% FBS and several hesperidin concentrations (10, 20, 50, or 100 μm). The hesperidin‐containing medium aliquots were prepared by diluting a hesperidin stock solution in DMSO for each test concentration with DMEM supplemented with 10% FBS. The final DMSO concentration was 0.5% in all experiments to avoid any cell damage by the solvent [14]. Pretreatment was performed for 1, 3, 6, 12, or 24 h at 37 °C in a humidified atmosphere with 5% CO_2_. Cells incubated without hesperidin were used as the control. Caco‐2 cells treated with hesperidin for 24 h were evaluated for viability using the WST‐8 cell proliferation reagent (Cell Counting Kit‐8, CCK‐8; Dojindo Molecular Technologies, Inc., Kumamoto, Japan).

### Fluorescein transport study and apparent permeability coefficient

Fluorescein transport experiments were performed in the hesperidin‐pretreated Caco‐2 cell monolayers using the Ussing Chamber system (Model U‐2500, Warner Instrument Co., Holliston, MA, USA). Caco‐2 cell monolayers in the transwell inserts were gently rinsed with Hanks's balanced salt solution (HBSS) buffer [pH 7.4, adjusted with 10 mm 4‐(2‐hydroxyethyl)‐1‐piperazine ethanesulfonic acid, HEPES] to remove any free test compound before carefully mounting the inserts in the Ussing Chamber. HBSS buffer aliquots (6.0 mL) were added to the apical and basolateral sides to equilibrate the monolayers for 10 min. The transport assay was started by replacing the apical buffer with fresh HBSS buffer containing 100 μm fluorescein. During the transport experiment, both sides were continuously bubbled with a mixture of O_2_:CO_2_ (95:5). Sample aliquots of 100 μL were collected from the basolateral side every 5 min for 30 min, and the same sample volume of fresh HBSS was simultaneously added to the basolateral side at each time point. The fluorescence of the collected samples was measured with a fluorescence spectrophotometer (Thermo Fisher Scientific, Waltham, MA, USA) at an excitation wavelength of 485 nm and an emission wavelength of 535 nm. The apparent permeability coefficient (P_app_) was calculated using the following equation:

$$P_{\mathrm{app}}\left(\mathrm{cm}/s\right)=\frac{V}{AC_{0}}\frac{dC}{dt}$$

where *V* is the assay solution volume in the basolateral compartment (6 mL); *A* is the surface area of the membrane (0.2826 cm^2^); *C*_0_ is the initial concentration in the apical compartment (mmol); d*C*/d*t* is the change in concentration in the basolateral compartment over time (mmol·s^−1^). The relative P_app_ of fluorescein in Caco‐2 cell monolayers pretreated with the test compound (hesperidin) was expressed as percentage (%) of the control P_app_.

### Real‐time reverse transcription PCR

To investigate the effects of hesperidin on the mRNA expression of TJ‐related genes and AMPK, Caco‐2 cells were cultured in 6‐well plates under the same cell culture conditions as described above. Total RNA was isolated from Caco‐2 cells using an RNeasy mini kit (Qiagen, Hilden, Germany) according to the manufacturer's instructions. RNA concentrations were measured with a Nanodrop spectrophotometer (Implen, Munich, Germany). Aliquots of 1 μg of total RNA were used as templates for reverse transcription reactions to synthesize cDNA with the AccuPower PCR premix (Bioneer, Daejeon, Korea) using the SimpliAmp Thermal cycler (Applied Biosystems, Foster City, CA, USA). The cDNA was amplified using 10 μL Power SYBR Green PCR Master Mix (Applied Biosystems), 2 μm of each primer specific for TJ‐related proteins (occludin, MarvelD3, JAM‐1, claudin‐1, claudin‐4, ZO‐1, and ZO‐2), AMPKα1 and β‐actin (Table 1), and 5.2 μL sterile deionized water in a final volume of 20 μL. Real‐time RT‐PCR was controlled by ABI StepOnePlus (Applied Biosystems) using a hot start at 95 °C for 10 min followed by 40 cycles of denaturing for 15 s at 95 °C, annealing for 60 s at 60 °C, and extension for 15 s at 72 °C. Expression values were normalized to the internal control gene, β‐actin, and the relative expression level in untreated cells was set to 1. All PCR reactions were performed in triplicate.

**Table 1:** Sequences of primers used for real‐time reverse transcription PCR.

Gene name	Primer sequence (5, −3)
Occludin	F: TGGCTGCTGCTGATGAATA
	R: CATCCTCTTGATGTGCGATAAT
MarvelD3	F: GAACCCCCTTCGGAGAGATA
	R: CGGCAAGGACAAAGTAGGAG
JAM‐1	F: CTGATCTTTGACCCCGTGAC
	R: ACCAGACGCCAAAAATCAAG
Claudin‐1	F: ACCGCTCAGGCCATCTAC
	R: CCAGCAGGATGCCAATTAC
Claudin‐4	F: GCTGGGAAGGGCAGTAGAG
	R: GGGCGTAATGGCAAGAGTAG
ZO‐1	F: GAATGATGGTTGGTATGGTGCG
	R: TCAGAAGTGTGTCTACTGTCCG
ZO‐2	F: GCTTTGGTGTGGACCAAGAT
	R: TCCATTATGGGTTTGCATGA
AMPKα1	F: GGGATCCATCAGCAACTATCG
	R: GGGAGGTCACGGATGAGG
β‐Actin	F: TGTTACCAACTGGGACGACA
	R: AAGGAAGGCTGGAAAAGAGC

### Western blot analysis

The expression levels of TJ‐related proteins in Caco‐2 cells treated with or without hesperidin (10 μm) for 6 and 24 h in the presence or absence of inhibitor (10 μm compound C as AMPK inhibitor, Sigma‐Aldrich) were detected by Western blot analysis. Cellular proteins were extracted using a radioimmunoprecipitation assay (RIPA) buffer (Sigma‐Aldrich) containing protease inhibitors (PhosSTOP; Roche, Basel, Switzerland), and the total protein concentration per sample was determined using the BCA protein assay kit (Thermo Fisher Scientific). Equal amounts of protein (15 μg per lane) per sample were loaded onto 4–12% Bis‐Tris bolt gels (Invitrogen, Waltham, MA, USA) for separation by electrophoresis. Then, the proteins were transferred onto a polyvinylidene difluoride (PVDF) membrane (Invitrogen), and the membrane was blocked with 5% non‐fat dried milk in TBS‐T (Tris‐buffered saline containing 0.05% Tween 20) for 1 h at room temperature. The membrane was incubated with primary antibodies for occludin (1:500, Santa Cruz Biotechnology, Inc., Dallas, TX, USA), JAM‐1 (1 : 500, Santa Cruz Biotechnology, Inc), claudin‐1, claudin‐4  (1 : 1000, Invitrogen), MarvelD3 (1 : 1000, Proteintech, Rosemont, IL, USA), or β‐actin (1 : 5000, Proteintech) overnight at 4 °C. Secondary antibodies (HRP‐conjugated goat anti‐rabbit IgG antibody or HRP‐conjugated horse anti‐mouse IgG antibody) were incubated (1 : 3000, Cell Signaling Technology, Danvers, MA, USA) for 1 h at room temperature. Immunoreactive proteins were detected on the membrane with the Pierce™ ECL Western blotting substrate (Thermo Fisher Scientific) using a western imaging system (CAS‐400SM, Davinch‐K, Seoul, Korea; Fig. S1). The amounts of TJ‐related proteins were quantified using the imagej software (NIH, Bethsda, MD, USA). Cellular protein levels were normalized based on the ratio of their levels to that of β‐actin. The normalized results were expressed as protein levels in hesperidin‐treated cells relative to those in the control cells without hesperidin treatment.

### Assay for phosphorylation of AMPK

Caco‐2 cells treated with or without 10 μm hesperidin for 6 and 24 h at 37 °C in a humidified atmosphere of 5% CO_2_ in air. Cells incubated in the medium without hesperidin were used as a control. For assay for phosphorylation, proteins were extracted from Caco‐2 cells using RIPA buffer containing PhosSTOP. The amounts of p‐AMPK and AMPK proteins were evaluated by Western blot analysis. All of the steps including electrophoresis, transfer, and blocking were obtained as mentioned above. The membrane was probed with a primary antibody for phospho (Thr^172^)‐AMPKα1 (1 : 1000, Merck Millipore, Burlington, MA, USA) overnight at 4 °C, and then incubated with the secondary antibody for 1 h at room temperature. The immunoreactive proteins on the PVDF membrane were analyzed with the Pierce™ ECL detection reagents using a western imaging system. For removing the p‐AMPK, the membrane was incubated in stripping buffer (Easter‐Blot Western Blot™, Biomax, Guri‐si, Korea) for 30 min, and then blocked with blocking buffer (Smart‐Block™, Biomax) at room temperature and reprobed with primary antibody for anti‐AMPKα1 (1 : 1000 dilution, Merck Millipore) overnight at 4 °C. Secondary antibody was incubated for 1 h at room temperature (Fig. S1). Quantification of p‐AMPK and AMPK were performed by the imagej software. Relative band intensities (p‐AMPK and AMPK) were normalized to the untreated cells, and the relative expression level in untreated cells was set to 1.

### Statistical analysis

All statistical analyses were performed using IBM® SPSS Statistics for Windows, version 21 (IBM Corp., Armonk, NY, USA). Results are expressed as the mean ± SEM. The statistical significance between groups was analyzed using one‐way ANOVA followed by the Tukey–Kramer *t*‐test for *post hoc* analysis at *P* < 0.05 to compare the differences among mean values. Other statistical evaluations were performed using the Student's *t*‐test.

## Results

### Cytotoxicity effect of hesperidin on Caco‐2 cells

**Fig. 1:**
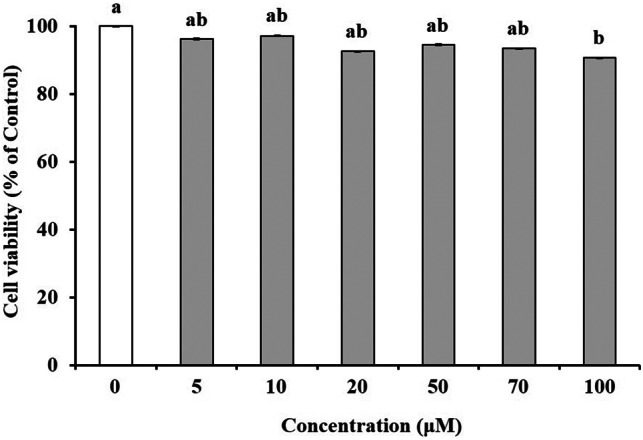
Effect of hesperidin pretreatment on Caco‐2 cell viability. Cell viability was determined after pretreating with 0 (Control), 5, 10, 20, 50, 70, and 100 μm of hesperidin for 24 h, respectively. Values are expressed as the mean ± SEM (*n* = 3). Different letters indicate the statistical differences at *P* < 0.05 between the groups by the Tukey–Kramer's *t*‐test.

We tested the cytotoxicity of hesperidin by assessing its effect on the viability of Caco‐2 cells using the CCK‐8 assay. For 24 h, Caco‐2 cells were incubated with different hesperidin concentrations ranging from 0 to 100 μm. Our results showed that Caco‐2 cells treated with 100 μm hesperidin had significantly lower cell viability than the untreated control cells. However, within the tested hesperidin concentration range, the viability of Caco‐2 cells remained above 90% (viability after treatment with 100 μm hesperidin: 91%), compared with that of untreated control cells (Fig. 1). Thus, all tested hesperidin concentrations could be used in our experiments because cell viability values above 90% were considered non‐cytotoxicity.

### Effect of hesperidin on the fluorescein transport across Caco‐2 cell monolayers

**Fig. 2:**
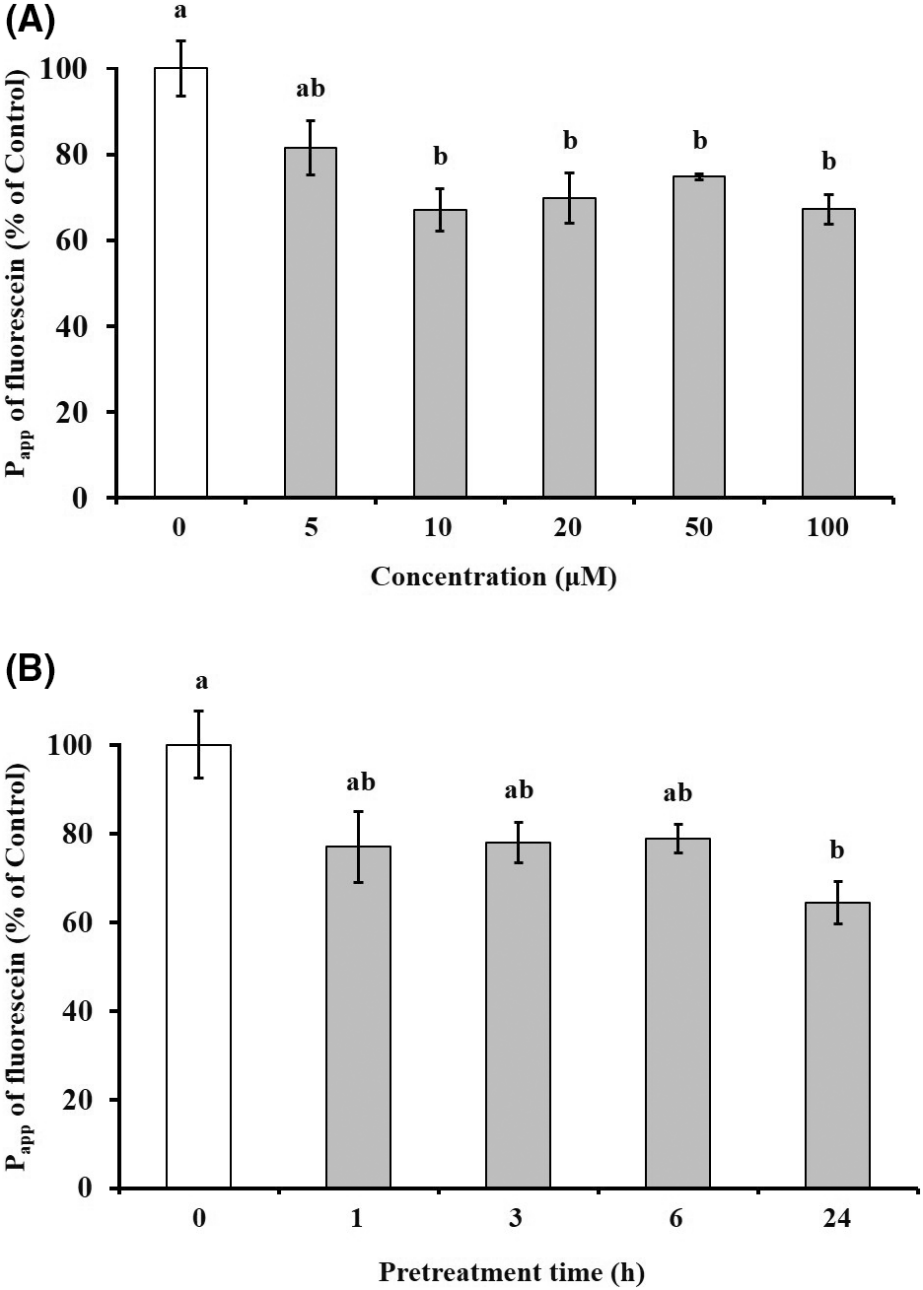
Effect of hesperidin on P_app_ values of fluorescein. Caco‐2 cell monolayers were pretreated with hesperidin at concentration of 5, 10, 20, 50, and 100 μm for 24 h (A). Time‐dependent effect (1, 3, 6, and 24 h) of hesperidin on fluorescein transport were investigated in 10 μm hesperidin pretreated with Caco‐2 cells (B). Values are expressed as the mean ± SEM (*n* = 5). Different letters indicate the statistical differences at *P* < 0.05 between the groups by the Tukey–Kramer's *t*‐test.

Fluorescein fluxes across Caco‐2 cell monolayers were measured to investigate the effect of hesperidin on the intestinal barrier function. Caco‐2 cell monolayers were pretreated with different hesperidin concentrations (5, 10, 20, 50, and 100 μm) for 24 h before the fluorescein transport experiments. As shown in Fig. 2A, the P_app_ of fluorescein was significantly lower in cell monolayers pretreated with > 10 μm hesperidin (5 μm: 7.2 ± 0.6× 10^−7^ cm·s^−1^; 10 μm: 6.0 ± 0.4 × 10^−7^ cm·s^−1^; 20 μm: 6.2 ± 0.5 × 10^−7^ cm·s^−1^; 50 μm: 6.7 ± 0.1 × 10^−7^ cm·s^−1^; 100 μm: 5.7 ± 0.3 × 10^−7^ cm·s^−1^) than in control cell monolayers without hesperidin pretreatment (control: 8.9 ± 0.6 × 10^−7^ cm·s^−1^). In addition, we performed a time‐course experiment (1, 3, 6, 24 h) to further assess the pretreatment effect of 10 μm hesperidin on the P_app_ of fluorescein (Fig. 2B). We observed a decrease in P_app_ over time, which was significant after 24 h of hesperidin pretreatment.

### Effect of hesperidin on TJ barrier integrity in Caco‐2 cell monolayers

**Fig. 3:**
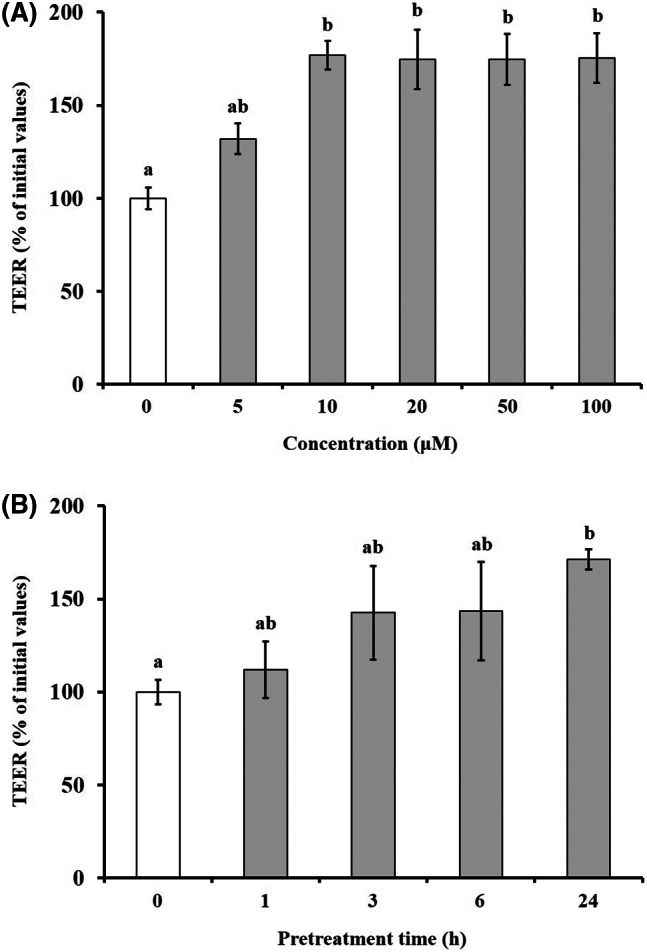
Effect of hesperidin on epithelial integrity. Transepithelial electrical resistance (TEER) was measured to confirm the intestinal epithelial integrity by hesperidin. Caco‐2 cell monolayers incubated with hesperidin at the concentration of 5–100 μm for 24 h (A). Caco‐2 cell monolayers treated with 10 μm hesperidin during 1, 3, 6, and 24 h (B). Values are expressed as the mean ± SEM (*n* = 5). Different letters indicate the statistical differences at *P* < 0.05 between the groups by the Tukey–Kramer's *t*‐test.

The TEER values of Caco‐2 cell monolayers were measured under various conditions to assess the tightness degree of the TJ structures in the presence of hesperidin. Specifically, the Caco‐2 cell monolayers were pretreated either with the hesperidin‐free medium as controls or with 5, 10, 20, 50, and 100 μm. As shown in Fig. 3A, pretreatment of the cell monolayers with > 10 μm hesperidin for 24 h significantly increased their TEER value. The time course with 10 μm hesperidin indicated an increase of the TEER value over time, and the highest value was reached after 24 h (Fig. 3B). These results suggested that the P_app_ reduction in the presence of hesperidin may be caused by a hesperidin‐induced enhancement of the intestinal barrier function (Table S1). Further experiments were performed in Caco‐2 cells pretreated with 10 μm hesperidin for 24 h.

### Effect of hesperidin on the expression of TJ‐related genes in Caco‐2 cells

**Fig. 4:**
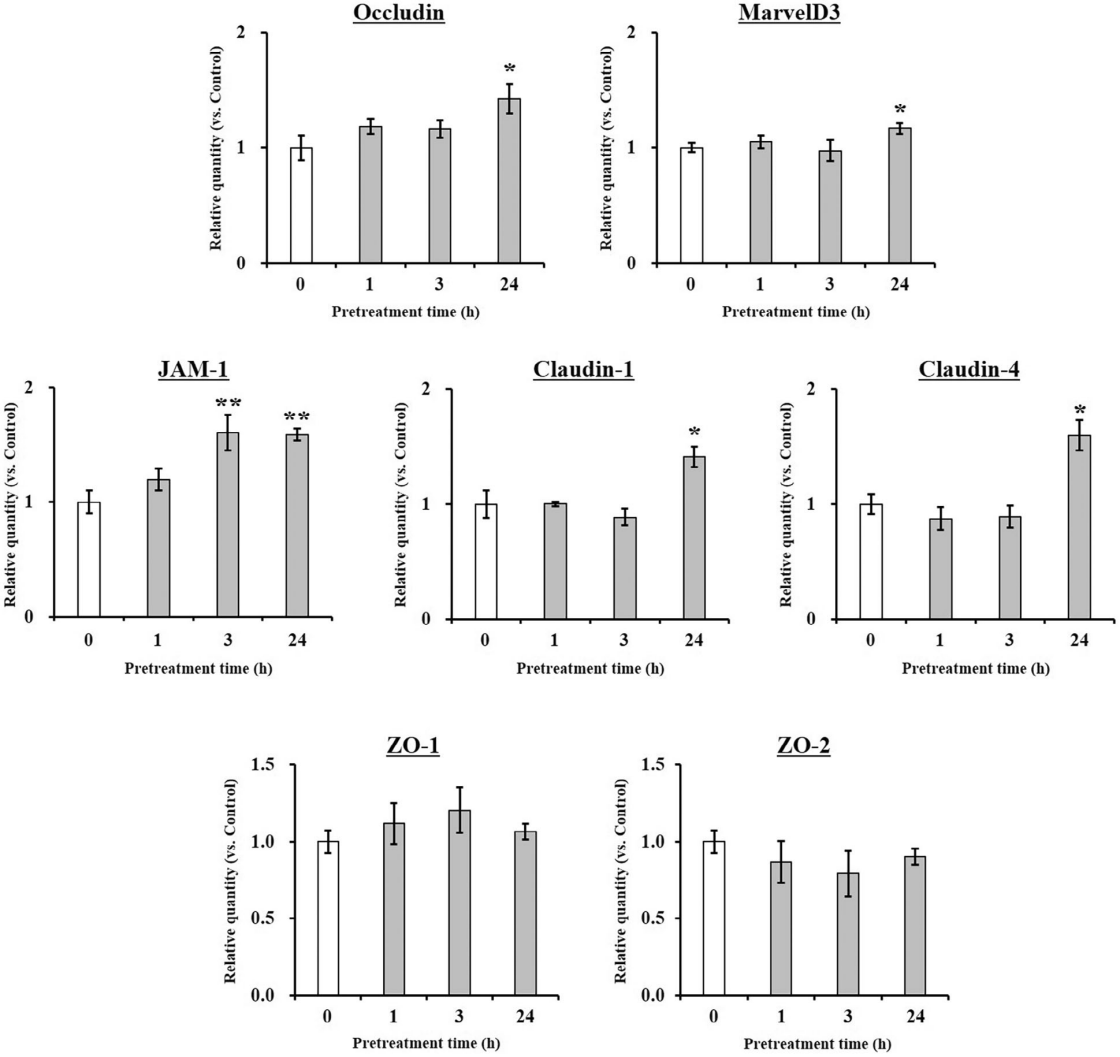
Effect of hesperidin on mRNA expression of TJ‐related genes in Caco‐2 cells. Caco‐2 cells were cultured with 10 μm hesperidin for 1, 3, and 24 h and calculated compared with untreated cells. Total RNA was isolated and the expression level of mRNA was measured by real‐time reverse transcription PCR using specific primers for occludin, MarvelD3, JAM‐1, claudin‐1, 4, ZO‐1, 2, and β‐actin. Values expressed as the mean ± SEM (*n* = 3). *P < 0.05 and **P < 0.01, significantly different from untreated Caco‐2 cells. Significant differences between 0 h (Control) and hesperidin‐pretreated groups were evaluated by the unpaired Student's *t*‐test.

**Fig. 5:**
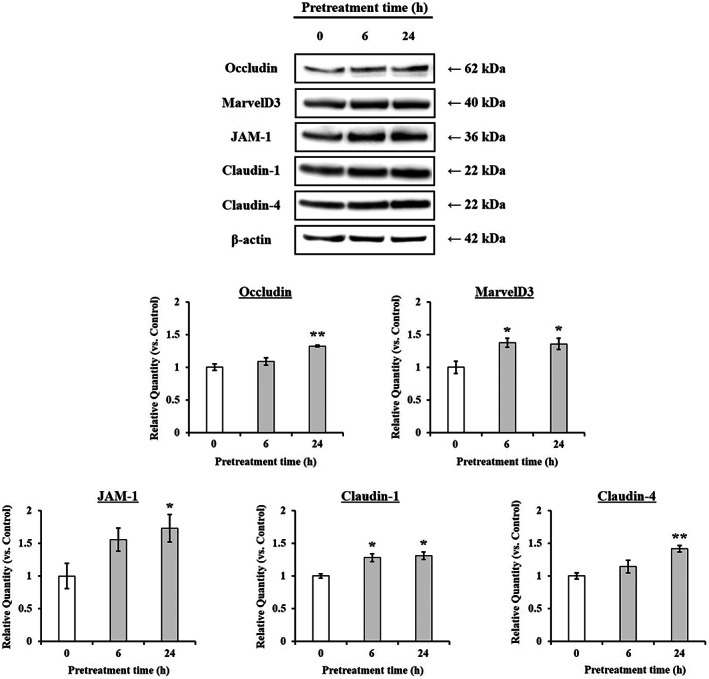
Effect of hesperidin on the expression of TJ‐related proteins in Caco‐2 cells. Caco‐2 cells were incubated with 10 μm hesperidin for 6, and 24 h. Specific bands for occludin, MarvelD3, JAM‐1, claudin‐1, 4, and β‐actin were quantitated by densitometric analysis. Values are expressed as the mean ± SEM (*n* = 3). *P < 0.05 and **P < 0.01, significantly different from untreated Caco‐2 cells. Significant differences between 0 h (Control) and hesperidin‐pretreated groups were evaluated by the unpaired Student's *t*‐test.

To clarify the mechanism underlying the inhibition of paracellular transport across Caco‐2 cell monolayers by hesperidin pretreatment, the expression of TJ‐related genes was investigated at mRNA and protein levels. Fig. 4 shows that Caco‐2 cells pretreated with 10 μm hesperidin had significantly higher mRNA levels of occludin, JAM‐1, claudin‐1, claudin‐4, and MarvelD3 than control cells (0 h pretreatment), whereas the ZO‐1 and ZO‐2 mRNA levels were not affected by the hesperidin pretreatment. Based on the mRNA expression results, the Western blot analysis was focused on occludin, JAM‐1, claudin‐1, claudin‐4, and MarvelD3, all of which are crucial for adjusting the permeability and tightness of the intestinal epithelium. As shown in Fig. 5, hesperidin‐pretreated cells had significantly higher occludin, JAM‐1, claudin‐1, claudin‐4, and MarvelD3 protein levels than the control cells. We specifically observed an early onset of the hesperidin‐induced protein level increase for claudin‐1 and MarvelD3 (6 h incubation), which indicated an effective enhancement of the TJ barrier function.

### Effect of the AMPK pathway in hesperidin‐pretreated Caco‐2 cells

**Fig. 6:**
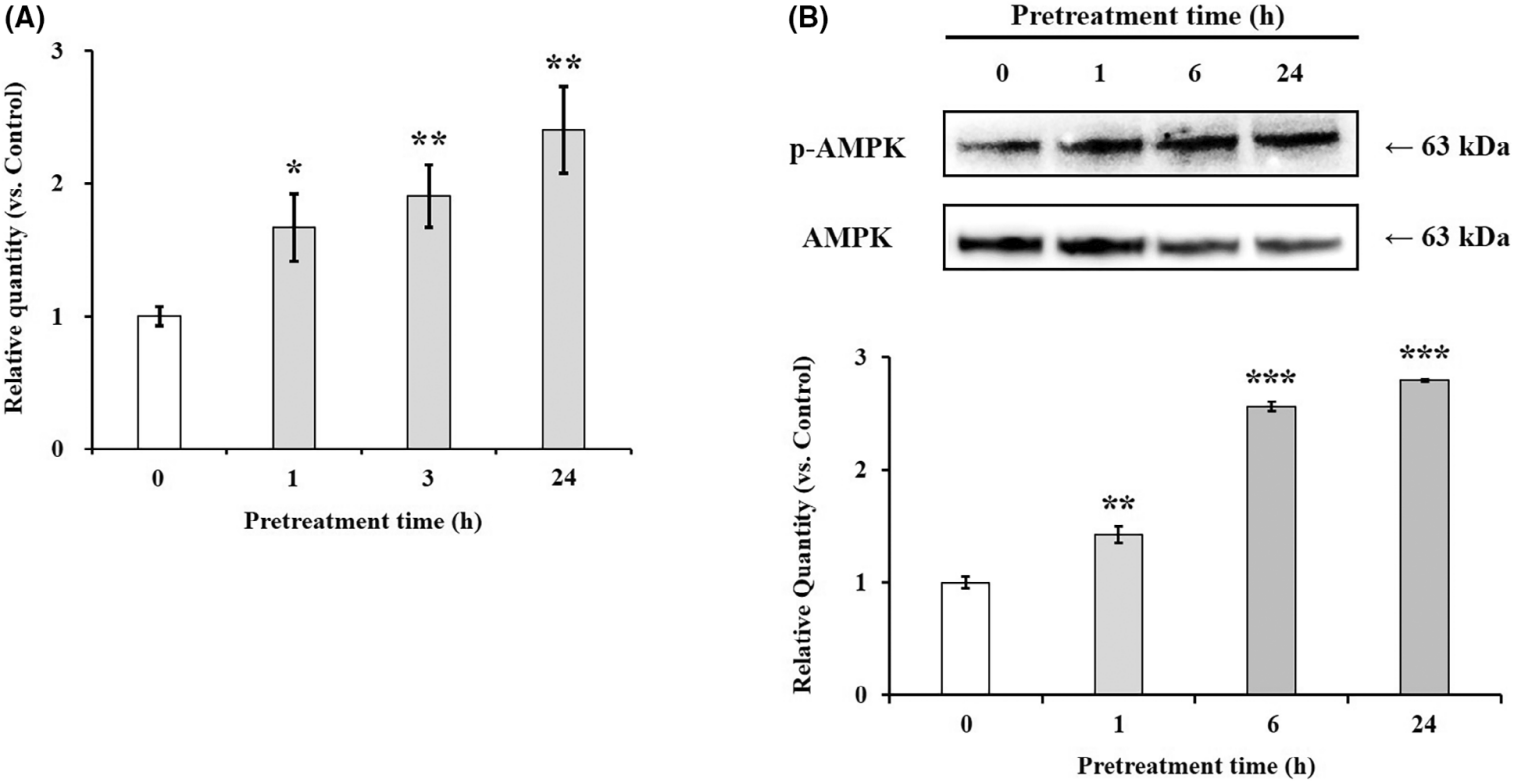
Effect of hesperidin on phosphorylation of AMPK. Caco‐2 cells were pretreated with or without 10 μm hesperidin for 1, 3, 6, and 24 h. Total RNA was isolated and the expression level of mRNA was measured by real‐time reverse transcription PCR using specific primers for AMPKα1 and β‐actin (A). Specific band for p‐AMPK and AMPK were quantified using densitometric analysis (B). Values are expressed as the mean ± SEM (*n* = 3). *P < 0.05, **P < 0.01, and ***P < 0.001 significantly different with untreated Caco‐2 cells. Significant differences between 0 h (Control) and hesperidin‐pretreated groups were evaluated by the unpaired Student's *t*‐test.

We then investigated the effect of hesperidin on the AMPK signaling pathway as a potential mechanism for the hesperidin‐associated enhancement of TJ protein expression. As shown in Fig. 6, AMPKα1 mRNA was significantly increased when Caco‐2 cells were treated with hesperidin for 1, 3, and 24 h, respectively. In addition, the phosphorylation level of AMPK (p‐AMPK) was higher in hesperidin‐treated cells than in control cells; this showed that hesperidin enhanced AMPK phosphorylation in Caco‐2 cells, suggesting that the mediation of the hesperidin‐associated effect on TJ protein expression possibly involved the activation of the AMPK signaling pathway.

### Effect of AMPK inhibitor on TJ‐related proteins in hesperidin‐pretreated Caco‐2 cells

**Fig. 7:**
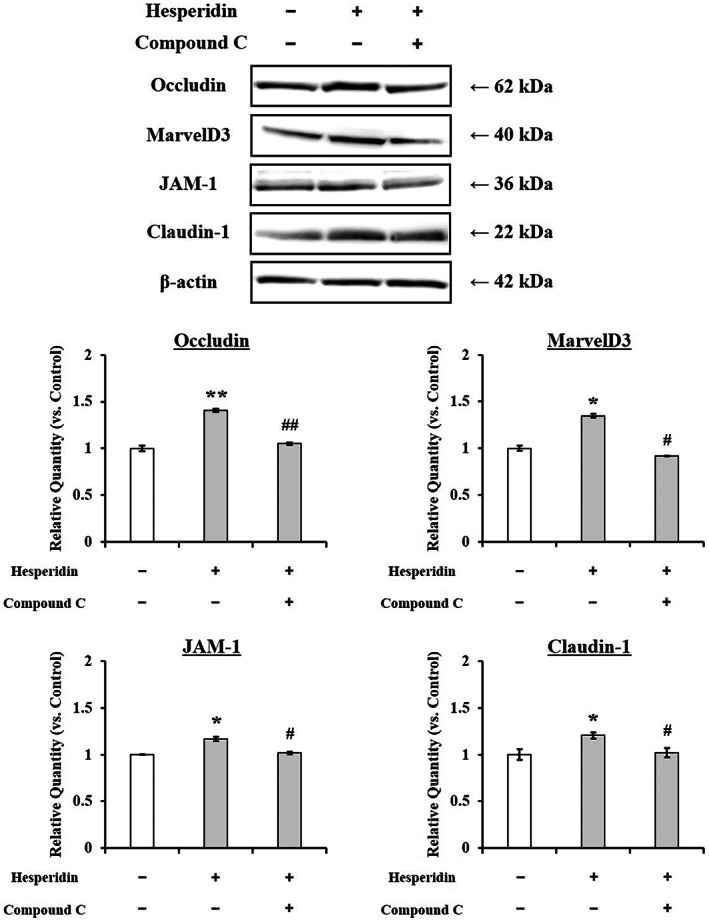
Effect of AMPK inhibition on TJ‐related proteins expression in Caco‐2 cells treated with hesperidin. Caco‐2 cells pretreated with 10 μm hesperidin in presence or absence of 10 μm compound C. Specific bands for occludin, MarvelD3, JAM‐1, claudin‐1, and β‐actin were quantitated by densitometric analysis. Values are expressed as the mean ± SEM (*n* = 3). *P < 0.05 and **P < 0.01, significantly different from untreated Caco‐2 cells. ^#^ *P* < 0.05 and ^##^ *P* < 0.01, significantly different from Caco‐2 cells treated with hesperidin. Significant differences between two groups were evaluated by the unpaired Student's *t*‐test.

To verify the involvement of AMPK activation on TJ barrier by hesperidin, TJ‐related protein expressions in Caco‐2 cells treated with hesperidin were observed in the presence of compound C, a specific inhibitor of AMPK. As shown in Fig. 7, hesperidin treatment for Caco‐2 cells resulted in a significant upregulation of TJ‐related proteins occludin, MarvelD3, JAM‐1, and claudin‐1 expression. In contrast, the reduction of the expression by compound C in Caco‐2 cells treated with hesperidin revealed that the upregulation of TJ‐related proteins, excepting claudin‐4, by hesperidin was responsible for AMPK activation.

## Discussion

Basic and clinical scientific studies have demonstrated the associations between intestinal barrier deficiencies and the pathogenesis of various gastrointestinal diseases [15, 16]. The intestinal barrier function depends on the interactions among various gut components, including the adhesive mucous gel layer, antibacterial peptides, and intercellular TJs. Among these components, the TJs constitute a major determinant of the intestinal barrier structure. The present study demonstrated that hesperidin treatment strengthened the intestinal barrier integrity in human intestinal Caco‐2 cell monolayers, a relevant intestinal epithelial cell model. The potentially beneficial effects of hesperidin treatment were indicated by increased TEER values and decreased fluorescein transport across the Caco‐2 cell monolayers. These effects were mediated via AMPK activation and the enhanced expression of TJ‐related proteins. The flavonoids in food are important polyphenols and are of great general interest due to their various bioactivity [17]. Many researches showed that flavonoid aglycones can easily permeate the epithelial cells due to their high lipophilicity and low molecular weight than flavonoid glycosides [10, 17, 18, 19, 20, 21, 22]. Zhang *et al*. [22] reported aglycones (quercetin and cyanidin) readily permeated into the intestinal mucosa within 2 h than their respective glycosides in Caco‐2 BBe1 cell model. Izumi *et al*. [20] also investigated that soy isoflavone aglycones (genistein, daidzein) were absorbed faster than their glucosides (genistin, daidzin) in humans. It also showed that hesperidin, glycoside of hesperetin, is poorly transported via paracellular space slower than hesperetin because of its hydrolytic action of microflora β‐glucosidases [10]. Interestingly, although aglycone is known as good intestinal permeability than their glycosides, the Majority of polyphenols exist as glycosides including glucoside, galactoside, rhamnoside, arabinoside, and rutinoside in plants [17, 23]. The most common polyphenols such as kaempferol, hesperidin, quercetin, cyanidin, genistin, daidzin, and delphinidin are predominantly in glycosidic forms [10, 20, 22]. Citrus fruits contain a tiny amount of hesperetin (hesperidin aglycone), and most of the flavanones exist as glycosides such as hesperidin (hesperetin‐7‐rhamnoglucoside) [10]. Specially, in terms of industrial cost, hesperidin is economically attractive because it can be easily isolated from the waste residues from citrus fruit processing. However, hesperetin can be obtained at a high cost because of modifying process hesperidin with the use of bacterial enzymes [24, 25, 26, 27]. Therefore, it is important to demonstrate the effect of glycosides of polyphenols, which have a comparative advantage in terms of price competitiveness. In addition, aglycone and its glycosides have similar biological effects, but not the same. Kim *et al*. [28] investigated that antioxidant activity of hesperidin and hesperetin, specially, hesperidin had stronger activity in protein oxidation than hesperetin, on the contrary, hesperetin had stronger activity in lipid oxidation than hesperidin. Hesperetin showed more toxicity than hesperidin in RAW 264.7 cells [29]. For TJ‐related genes, aglycone has similar or more powerful biological effects compared with its glycosides. Nakashima *et al*. [30] investigated that naringenin (aglycone) upregulates some claudins (claudin‐1, ‐3, and ‐7), whereas narirutin (glycosides of naringenin) did not show any remarkable change in the TJs on the MDCK II cells. As reported by Noda *et al*. [31] hesperetin increases the occludin, claudin‐1, and − 4 whereas did not affect ZO‐1, ‐2, JAM‐1, and claudin‐3. These findings are very similar and support well our present data (Figs 4 and 5). In this study, we demonstrated that hesperidin diminished fluorescein paracellular transport via the TJ route (Figs 2 and 3). When the Caco‐2 cell monolayers were preincubated with < 100 μm hesperidin for 24 h, cell viability remained above 90%, indicating that the effect was non‐cytotoxic (Fig. 1). We concluded that the hesperidin‐mediated inhibition of the fluorescein transport could have been induced by a change in associated signaling pathways, causing the blockage of the TJ‐mediated transport route without a toxic effect. Our results showed that hesperidin activated AMPK, which strengthened the TJ barrier and stimulated TJ‐related gene expression (Figs 4, 5, 6). More specifically, hesperidin increased the mRNA and protein levels of several TJ components, including occludin, MarvelD3, JAM‐1, claudin‐1, and claudin‐4, but it did not affect ZO‐1 and ZO‐2 mRNA levels (Figs 4, 5, 6). There are many reports that indicate a major role for several TJ‐related proteins, including occludin, MarvelD3, JAM‐1, and claudins, in the regulation of paracellular permeability and the assembly of TJ structures [4, 13, 32]. Specifically, occludin is involved in the regulation of the paracellular pathway for small molecule diffusion. JAM proteins are known to regulate the epithelial barrier function associated with paracellular permeability and TJ assembly. The claudin proteins function as the structural backbone of TJs; moreover, claudin‐1 and ‐4 can decrease paracellular permeability by tightening the TJs in the intestine [32, 33, 34]. It has been reported that MarvelD3 intracellularly interacts with occludin and claudins, including claudin‐1 and ‐4 [35, 36]. TEER is widely used as a strong indicator for assessing the dynamics of the TJ integrity in cell culture models of epithelial monolayers [37]. Otani *et al*. [38] reported that the TEER value was lower in claudin gene‐knockout cell lines than in normal cell lines, whereas the overexpression of claudin‐1 and ‐4 was associated with an increase in TEER [38, 39]. In addition, JAM‐1 inhibition by calcium depletion also prevents the recovery of TEER in epithelial monolayers [32, 40]. These results suggest that JAM‐1 and the claudin proteins, especially claudin‐1 and ‐4, are essential for establishing and maintaining the permeability barrier, which is associated with the TEER value. In our study, we also found that the hesperidin‐mediated increase of the TEER value (Fig. 3) was associated with an elevated expression of JAM‐1, claudin‐1, and claudin‐4 (Figs 4 and 5). In Caco‐2 cells pretreated with hesperidin for 24 h, the TEER value increased throughout the entire incubation period and, according to the RT‐PCR and immunoblot analyses of TJ‐related proteins, occludin, MarvelD3, JAM‐1, claudin‐1, and claudin‐4 appear to be responsible for the observed TEER increase. Many other studies show that an enhanced intestinal barrier function depended on the modulation of TJ‐related protein expression by certain signaling pathways [13, 32, 41]. The AMPK signaling pathway modulates various important membrane transport proteins, and activated AMPK is a prerequisite for regulating the expression of TJ proteins, including occludin, claudin‐1, and ZO‐1 [13, 42]. Here, we demonstrated that hesperidin‐stimulated AMPK phosphorylation (Fig. 6), suggesting that hesperidin‐stimulated expression of TJ genes in Caco‐2 cells was regulated by AMPK activation. In addition, we found that AMPK activation is possible involvement of TJ‐related genes occludin, MarvelD3, JAM‐1, and claudin‐1 by hesperidin (Fig. 7), but no link with the claudin‐4 expression (data not shown). These findings clearly showed that hesperidin activated AMPK signaling and strengthened the intestinal barrier function linked to TJ‐related proteins occludin, MarvelD3, JAM‐1, and claudin‐1. However, it is still unknown how hesperidin‐induced phosphorylation of AMPK is controlled by upstream signaling pathways that are triggered by other kinases, including liver kinase B1 (LKB1), Ca^2+^/calmodulin‐dependent protein kinase kinase (CaMKK), and transforming growth factor‐β‐activated kinase (TAK‐1) [43, 44]. We are currently continuing our work by testing the LKB1 and CaMKK expression in the presence of hesperidin using RT‐PCR and Western blotting to determine the involvement of upstream pathways (data not shown). Thus, further experiments are needed to clarify the regulation of upstream signals by hesperidin in Caco‐2 cells.

## Conclusions

The results of the present study demonstrated that hesperidin effectively enhanced the intestinal barrier function by stimulating the AMPK‐mediated expression of occludin, MarvelD3, JAM‐1, and claudin‐1 proteins. Our findings are well represented about the enhancement effect of hesperidin on human intestinal barrier function and its related mechanism mediated by the AMPK signaling pathway. Based on our results, we conclude that hesperidin is potentially effective in improving intestinal barrier function.

## Conflict of interest

The authors declare no conflict of interest.

## Author contributions

HYP involved in writing—original draft, review and editing, visualization, supervision, project administration, methodology, and investigation; JHY involved in methodology, investigation, and formal analysis.

## Supporting information


**Fig. S1.** Full blots for fig. 5 (A), fig. 6 (B), and fig. 7 (C).
**Fig. S2.** TEER value monitoring of Caco‐2 cell monolayers for cultivation days. Caco‐2 cells seeded at a density of 4 × 10^5^ cells·mL^−1^ were grown in a cell culture insert coated with type I collagen. For 48 h, the cells were cultured in a basal medium for seeding with MITO+™ serum extender as a supplement. Then, the medium was changed to enterocyte differentiation medium containing MITO+™ serum extender, and incubation was continued for 72 h to form monolayers.
**Table S1.** Effect of hesperidin on real TEER values. Transepithelial electrical resistance (TEER) was measured to confirm the intestinal epithelial integrity by hesperidin. Caco‐2 cell monolayers incubated with hesperidin at the concentration of 5 to 100 μm for 24 h (A). Caco‐2 cell monolayers treated with 10 μm hesperidin during 1, 3, 6, and 24 h (B). Values are expressed as the mean ± SEM (n = 5).

## Data Availability

The data that support the findings of this study are available from the corresponding author upon reasonable request.
