# Supplementary material for: Hesperidin enhances intestinal barrier function in Caco‐2 cell monolayers via AMPK‐mediated tight junction‐related proteins
Source: FEBS Open Bio. 2023 Feb 7;13(3):532–44. doi: 10.1002/2211-5463.13564 (PMC9989920; doi:10.1002/2211-5463.13564)
Supplement: Supplementary file 1 — Fig. S1. Full blots for fig. 5 (A), fig. 6 (B), and fig. 7 (C). Fig. S2. TEER value monitoring of Caco‐2 cell monolayers for cultivation days. Caco‐2 cells seeded at a density of 4 × 10^5^ cells·mL−1 were grown in a cell culture insert coated with type I collagen. For 48 h, the cells were cultured in a basal medium for seeding with MITO+™ serum extender as a supplement. Then, the medium was changed to enterocyte differentiation medium containing MITO+™ serum extender, and incubation was continued for 72 h to form monolayers. Table S1. Effect of hesperidin on real TEER values. Transepithelial electrical resistance (TEER) was measured to confirm the intestinal epithelial integrity by hesperidin. Caco‐2 cell monolayers incubated with hesperidin at the concentration of 5 to 100 μm for 24 h (A). Caco‐2 cell monolayers treated with 10 μm hesperidin during 1, 3, 6, and 24 h (B). Values are expressed as the mean ± SEM (n = 5). [file FEB4-13-532-s001.docx]

**Fig. S1 Park *et al.***

**
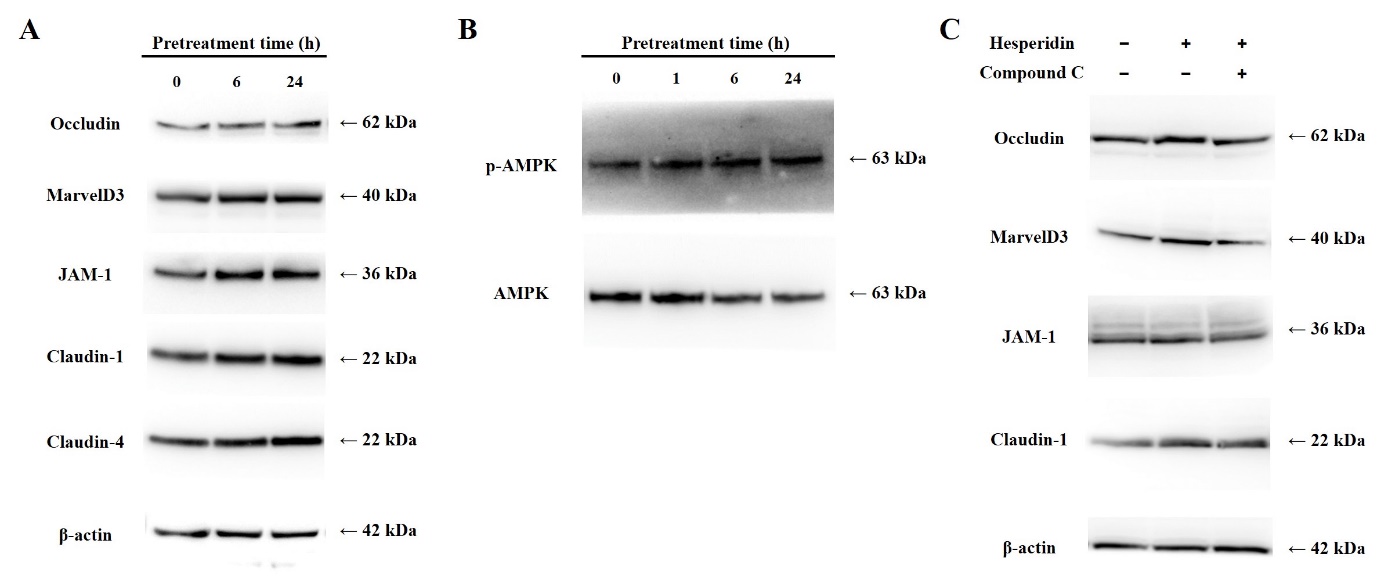
**

**Supplemental Figure S1.** Full blots for figure 5 (A), figure 6 (B), and figure 7 (C)

**Fig. S2 Park *et al.***

**
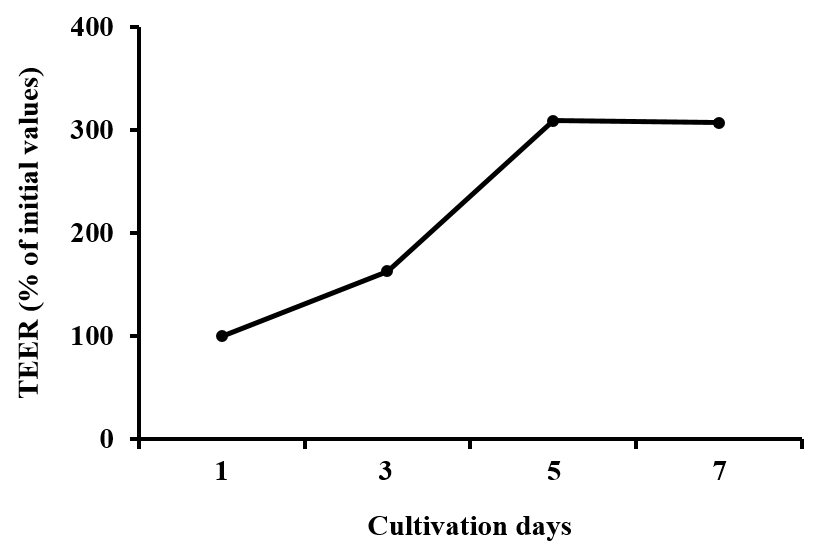
**

**Supplemental Figure S2. TEER value monitoring of Caco-2 cell monolayers for cultivation days.** Caco-2 cells seeded at a density of 4 × 10^5^ cells/mL were grown in a cell culture insert coated with type I collagen. For 48 h, the cells were cultured in a basal medium for seeding with MITO+^TM^ serum extender as a supplement. Then, the medium was changed to enterocyte differentiation medium containing MITO+^TM^ serum extender, and incubation was continued for 72 h to form monolayers.

**Table S1 Park *et al.***

**A**

| **Control** | **Concentration (μM)** | | | | |
| --- | --- | --- | --- | --- | --- |
|  | **5** | **10** | **20** | **50** | **100** |
| 340 ± 19.65 | 461 ± 28.10 | 586 ± 26.18 | 594 ± 54.37 | 593 ± 46.87 | 596 ± 45.64 |

**B**

| **Control** | **Pretreatment time (h)** | | | |
| --- | --- | --- | --- | --- |
|  | **1** | **3** | **6** | **24** |
| 341 ± 22.57 | 382 ± 51.93 | 486 ± 85.39 | 489 ± 89.65 | 584 ± 18.35 |

**Supplemental Table S1. Effect of hesperidin on real TEER values.** Transepithelial electrical resistance (TEER) was measured to confirm the intestinal epithelial integrity by hesperidin. Caco-2 cell monolayers incubated with hesperidin at concentration of 5 to 100 μM for 24 h (A). Caco-2 cell monolayers treated with 10 μM hesperidin during 1, 3, 6, and 24 h (B). Values are expressed as the mean ± SEM (n = 5).
